# Emotion Knowledge, Theory of Mind, and Language in Young Children: Testing a Comprehensive Conceptual Model

**DOI:** 10.3389/fpsyg.2019.02144

**Published:** 2019-09-19

**Authors:** Elisabetta Conte, Veronica Ornaghi, Ilaria Grazzani, Alessandro Pepe, Valeria Cavioni

**Affiliations:** “R. Massa” Department of Human Sciences for Education, University of Milano – Bicocca, Milan, Italy

**Keywords:** theory of mind, emotion knowledge, language, toddlers, preschoolers, structural equation modeling

## Abstract

Numerous studies suggest that both emotion knowledge and language abilities are powerfully related to young children’s theory of mind. Nonetheless, the magnitude and direction of the associations between language, emotion knowledge, and theory-of-mind performance in the first years of life are still debated. Hence, the aim of this study was to assess the direct effects of emotion knowledge and language on theory-of-mind scores in 2- and 3-year-old children. A sample of 139 children, aged between 24 and 47 months (*M* = 35.5 months; *SD* = 6.73), were directly administered measures of emotion knowledge, theory of mind, and language. We conducted structural equation modeling (SEM) to evaluate the effects of these variables within a single comprehensive framework, while also controlling for any effects of age and gender. The proposed structural equation model provided an excellent fit for the data, indicating that both children’s emotion knowledge, and their language ability had direct positive effects on theory of mind scores. In addition, age was found to wield statistically significant effects on all the variables under study, whereas gender was not significantly associated with any of them. These findings suggest the importance of fostering young children’s emotion knowledge and language ability with a view to enhancing their comprehension of mental states.

## Introduction

Early childhood is a critical period for acquiring the skills needed to interact successfully with others and construct positive peer relationships. Starting in infancy, children are stimulated to familiarize themselves with the workings of the human social world and meet the many challenges of everyday situations by thinking socially. This involves gradually learning to represent themselves and others as individuals with inner states, such as intentions, desires, beliefs, and emotions.

Acquiring the ability to reason about others’ mental states bears social advantages, both in childhood and later in the development, enhancing popularity, peer acceptance, and the capacity to maintain friendships over time (Slaughter et al., 2015; Peterson et al., 2016). Children who are better at mind-reading also receive higher scores on measures of prosocial behavior (Imuta et al., 2016; Conte et al., 2018). Moreover, research findings suggest that positive relationships with peers may in turn directly and indirectly enhance school readiness and later academic performance (Lagattuta et al., 2014; Lecce et al., 2014, 2015). The benefits of possessing mindreading skills are not limited to social and academic outcomes. Indeed, a number of studies have found that young children’s understanding of others’ minds is closely related to their development of emotional competence (Denham, 1986; Ensor and Hughes, 2008; O’Brien et al., 2011) and language abilities (Curby et al., 2015; Imuta et al., 2016; Strand et al., 2016; Conte et al., 2018). Research on mental state talk has confirmed this connection by showing that conversations about inner states promote social cognitive skills (Meins et al., 2013; Ornaghi et al., 2015; Grazzani et al., 2016b).

However, the associations between the ability to reason about others’ minds, knowledge of emotions, and language abilities need to be further explored in relation to early childhood, a period when these skills undergo strong development and may be fostered by educational activities. We now take an in-depth look at these three main constructs and their reciprocal associations in order to build up a rationale for the present study.

### Emotion Knowledge in Early Childhood

Recent studies have shown that children’s emotion knowledge comprises two distinct dimensions, namely emotion recognition and emotion situation knowledge (Bassett et al., 2012; Sette et al., 2015). Recognizing emotions means that the child is able to label facial expressions using emotion lexicon (expressive emotion knowledge), as well as to identify emotions when they are expressed with verbal labels (receptive emotion knowledge). Emotion situation knowledge is the ability to reflect upon situational and contextual cues of emotion, both stereotypical and non-stereotypical. Although these two dimensions of emotion knowledge are separate, they are related to one another to some degree. Specifically, children’s ability to recognize emotions precedes and forms the basis for their knowledge of reactions to emotion-eliciting situations, and both of these acquisitions proceed concurrently with overall cognitive and linguistic development (Sette et al., 2015).

This pattern of interrelationships is evident in the emotional development trajectory spanning infancy and the preschool years. First, early in life, infants begin to recognize and differentiate between the facial expressions of others (Trevarthen, 2011). Between 9 and 24 months, they learn to discriminate between positive and negative emotions (Otte et al., 2015; Walle et al., 2017), recognizing positive emotions more easily than negative ones (Widen and Russell, 2008). At around 18–24 months they acquire the terms required to label basic emotions – initially “happy,” “angry,” and “sad,” followed by “scared,” “surprised,” and “disgusted” (Widen and Russell, 2003). Interestingly, Widen and Russell (2010) found that children’s verbal categorization of emotions develops based on two initial broad categories, that is to say, positive and negative emotions; between the ages of 2 and 4 years, children gradually expand their vocabulary and learn to fully differentiate between the emotions within each of the two macro-categories.

During the preschool years, children also begin to understand that emotions are experienced due to situational factors, and again positive situational cues are identified earlier than negative ones (Bassett et al., 2012). Moreover, situations eliciting sadness and anger are learned before those eliciting fear (Denham and Couchoud, 1990; Widen and Russell, 2008).

Regarding individual differences, studies have found that children’s emotional competence varies as a function of gender. Specifically, girls often score higher on tasks measuring emotion knowledge (Dunn et al., 1991; Denham et al., 2002; Bassett et al., 2012) and are better than boys at identifying emotions in stereotypical situations (Sette et al., 2015). Although these findings suggest greater intrinsic emotional skills in females, this may be caused by gender-related socialization practices, in that caregivers typically engage girls more frequently than boys in conversation about emotions (Denham et al., 2010). The contribution of parental practices may explain why other studies have identified few gender differences in children’s emotion knowledge (Grazzani et al., 2016a; Fidalgo et al., 2017; Conte et al., 2018).

### The Development of Theory of Mind in Early Childhood

Acquisition of a theory of mind – that is to say, the understanding that people have mental states such as intentions, desires, beliefs which guide their actions – is a key milestone in children’s development of social cognition (Wellman, 1990). Contrary to what was believed in the past, a growing body of research attests that very young children, from approximately 12 to 18 months of age, are implicitly aware of other people’s mental states (Low and Perner, 2012) and are able to recognize that people have intentions, goals, desires, beliefs, and even false beliefs which guide their actions (Onishi and Baillargeon, 2005; Scott and Baillargeon, 2009; Yott and Poulin-Dubois, 2016).

Nevertheless, it is only during their later acquisition of language abilities that children progress to an explicit understanding of inner states. Wellman (2014, 2018) proposed that the development of theory of mind may be viewed as a five-step process. First, by age 2 years, children grasp that different people, including themselves, can have different desires. Second, at around 3 years of age, they come to understand the concepts of true belief and differing beliefs. Next, at around 4 years of age, they realize that knowledge is only possible if an individual has access to information. The fourth step, at around 4-5 years, involves developing the understanding that even when a piece of information is true, it is possible for someone to falsely believe something else. Finally, at age 6 years, children become aware of hidden emotions, realizing that an individual can feel one emotion yet display another.

Typical children may vary in terms of the exact ages at which they complete each of these steps in their theory-of-mind development. Such discrepancies may be a function of biological and intrinsic factors, such as gender. Indeed, while most studies have identified no gender differences in young children’s mind-reading (Eggum et al., 2011; Wu and Su, 2014; Conte et al., 2018), some have found that girls perform better than boys on theory-of-mind tasks (Charman et al., 2002; Slaughter et al., 2015). Hence, the relationship between gender and theory of mind remains a topic in need of further investigation.

### The Association Between Emotion Knowledge and Theory of Mind

Studies in developmental psychology have gathered a large body of evidence for strong positive associations between children’s knowledge of emotions and their understanding of others’ minds (Cutting and Dunn, 1999; de Rosnay et al., 2004; Harwood and Farrar, 2006). Significant positive associations were reported with 2- and 3-year-old children (Denham, 1986; Grazzani et al., 2016a), as well as with preschoolers (Bulgarelli and Molina, 2016; Ornaghi et al., 2016), and these correlations remained significant after controlling for age.

Alongside these correlational studies, research examining the direction of the relationship between emotion knowledge and theory of mind has also yielded interesting, yet contradictory, results. On the one hand, some scholars have found support for the hypothesis that theory of mind skills predict subsequent individual differences in emotion knowledge. For instance, Seidenfeld et al. (2014) administered measures of theory of mind and emotion knowledge to at-risk children aged from 3 to 5 years, repeating them one year later. The authors reported that false belief comprehension at Time 1 explained 10% of children’s emotion knowledge at Time 2. Similar results were obtained by Hughes and Dunn (2002) in a longitudinal study with a slightly older sample, aged 3–4 years at the first time point and 6–7 years at the second. In this case, correlational analyses showed that children’s earlier theory of mind performances were related to their later explanations of emotion causes.

On the other hand, research has also yielded evidence that preschoolers’ understanding of emotions shapes their understanding of others’ inner states (e.g., Hughes and Dunn, 1998; Weimer et al., 2012). In an attempt to clarify the direction of causality at play in between these two variables, O’Brien et al. (2011) conducted a longitudinal study designed to test three hypotheses, namely that emotion knowledge predicts theory of mind, that theory of mind predicts emotion knowledge, and that the development of the two variables proceeds independently. The authors assessed social cognitive skills when children were 3 years old and again 1 year later. Hierarchical multiple regression analyses revealed that emotion knowledge at age 3 predicted theory of mind at age 4, but not the reverse. Therefore, given that children learn about emotions prior to understanding others’ thinking, it is likely that recognizing emotions is easier than identifying cognitive mental states.

Similar findings emerged from a longitudinal study carried out by Eggum et al. (2011). The researchers found that children’s understanding of emotions at 42 months predicted their theory-of-mind performances at 54 and 72 months. Interestingly, however, theory of mind skills at 54 months predicted emotion comprehension at 6 years of age.

Overall, these findings suggest the possibility that in younger children the ability to understand emotions may foster reasoning about cognitive states, which in turn facilitates the acquisition of emotional skills as the child continues to develop.

### The Role of Language in the Association Between Emotion Knowledge and Theory of Mind

The acquisition of both emotion knowledge and theory of mind is related to the development of language abilities. The first evidence for a strong association between social cognitive skills and language abilities comes from children’s everyday use of psychological lexicon. In fact, at approximately 2 years of age children begin to use terms indicating inner states (Bartsch and Wellman, 1995) and the more children’s language skills develop, the better they are able to put their own and others’ feelings and mental states into words (Harris et al., 2016). Children’s first references to internal states concern emotions (e.g., “happy,” “angry,” and “sad”), followed by the use of terms describing cognitive states (such as “know,” “think,” and “believe”) from approximately 3 years of age (Wellman, 2014). Further evidence of the key role of language abilities in the development of social cognition comes from studies involving children with autism, hearing, language impairments, and delays. In fact, children with language difficulties perform more poorly on tasks that require understanding of others’ emotions, intentions, and thoughts (Nelson et al., 2011; Slaughter and Peterson, 2011; Peterson et al., 2012; Broekhof et al., 2015; Cavioni et al., 2017; Rieffe and Wiefferink, 2017).

A number of studies have shown that children’s advanced language abilities impact on their knowledge about emotions (de Rosnay et al., 2004; Vallotton and Ayoub, 2011; Grazzani et al., 2019). However, other research has pointed up reciprocal effects between language and emotion knowledge. For example, Ornaghi et al. (2019) found that toddlers’ language ability was significantly correlated with their emotion knowledge scores. In another study, Strand et al. (2016) investigated the relations between emotion understanding and receptive language skills in children aged between 36 and 67 months: Their structural equation modeling (SEM) analyses revealed bidirectional associations between the variables for older children only (49–67 months).

Strong positive associations have also been found between language abilities and theory of mind (Meins et al., 2013; Imuta et al., 2016). Furthermore, language development has been found to predict the acquisition of theory of mind (Astington and Jenkins, 1999; Ruffman et al., 2002; San Juan and Astington, 2017). A meta-analysis by Milligan et al. (2007) explored the relationship between language and false-belief understanding in children younger than 7 years. In addition to finding significant positive correlations, after controlling for age, the authors identified bidirectional influences between the two variables. However, language ability had a stronger influence on theory of mind than vice versa.

Further key insights are offered by studies that assessed emotion knowledge, theory of mind, and language within a single comprehensive framework. For example, Bulgarelli and Molina (2016) administered emotion understanding, theory of mind, and language tasks to 118 preschoolers. Their regression analyses showed that language wielded both direct and indirect effects on the other variables. Similarly, Grazzani et al. (2018) carried out a cross-sectional study involving 389 children aged 3–8 years. Three main findings emerged: all the variables under study were significantly and positively correlated; children’s emotion comprehension and language explained variance in their theory of mind performance, independently of age and gender; language mediated the relationship between emotion understanding and theory-of-mind skills.

Thus, overall the existing literature suggests that language abilities are strongly associated with social cognition skills and may affect them. Nevertheless, to the best of our knowledge, the existing studies are lacking in two respects. First, cross-sectional studies investigating the associations between these variables have often used correlational or regression analyses rather than SEM and therefore did not provide detailed information about the direct and indirect effects among variables (Schreiber et al., 2006; Raykov and Marcoulides, 2012). Second, most studies on these variables have been carried out with preschoolers or older children, despite the fact that the acquisition of emotion knowledge, theory of mind, and language begins during toddlerhood and even earlier. Thus, testing these variables within a single comprehensive framework and with a sample of young children may help to clarify whether and to what extent emotion knowledge, theory of mind, and language are associated with one another at the early stages of development. The aim of the current study was to address these gaps in the literature.

### The Present Study

We set out to advance existing knowledge concerning the patterns of association among emotion knowledge, theory of mind, and language in the early years, also controlling for age and gender. While language is known to be strongly associated with children’s acquisition of both emotion knowledge (de Rosnay et al., 2004; Vallotton and Ayoub, 2011; Strand et al., 2016) and understanding of others’ minds (Astington and Jenkins, 1999; Ruffman et al., 2002; Milligan et al., 2007; San Juan and Astington, 2017), the cumulative relationships among these variables have never been explored with younger children within a single comprehensive framework.

**FIGURE 1:**
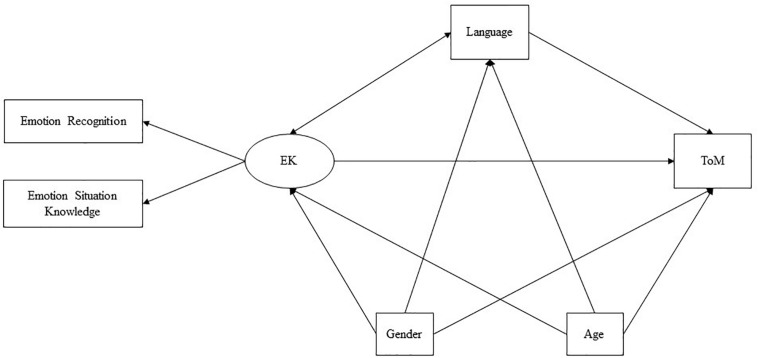
Conceptual model of estimated patterns of association between demographic characteristics, emotion knowledge, language, and theory of mind.

We decided to test a model with theory of mind as a latent target variable, analyzing the direct effects of emotion knowledge on theory of mind as well as the indirect effects via language ability. The decision to examine this direction of influence between emotion knowledge and theory of mind was underpinned by multiple criteria. First, infant research has consistently identified a developmental trajectory whereby children seem to display basic emotion comprehension abilities before going on to acquire initial mind-reading skills. In fact, the ability to discriminate among and recognize emotions, as well as to imitate their expression, appears during first months of life (see Widen and Russell, 2008; Trevarthen, 2011; Otte et al., 2015; Walle et al., 2017), whereas early implicit ability to attribute cognitive states (e.g., intentions, goals, desires, and beliefs) appears later, at around 14–18 months (Onishi and Baillargeon, 2005; Scott and Baillargeon, 2009; Low and Perner, 2012; Yott and Poulin-Dubois, 2016). The notion that theory of mind develops later than emotion understanding is further borne out by research on psychological lexicon. In fact, children’s use of emotional state talk predates their initial references to beliefs and other cognitive mental states (see Grazzani et al., 2019). Emotion terms appear as early as the child begins to talk, whereas cognitive terms become a stable feature of the child’s lexicon at the age of approximately 3–4 years (Bartsch and Wellman, 1995; Wellman, 2014). Second, some previous studies have suggested that emotion knowledge contributes to the development of theory of mind skills in young children (e.g., Hughes and Dunn, 1998; Eggum et al., 2011; O’Brien et al., 2011). Although still other studies have yielded evidence for the opposite influence, these were focused on older children and on theory of mind tasks different to those investigated in the present study. As some scholars have suggested, the direction of associations between emotion knowledge and theory of mind may depend on children’s developmental age and the specific skills being tested (see Eggum et al., 2011; Weimer et al., 2012; Seidenfeld et al., 2014). Hence, the age of the children in our sample (2 and 3 years old) and the skills under study informed our decision to test a model in which emotion knowledge was expected to wield a direct effect on theory of mind performance. More specifically, we set out to test the following research hypotheses: First, in line with the current literature, we expected to find statistically significant associations among the variables under study, even after controlling for participants’ demographic characteristics. The variables age and gender were included in the model to be assessed. Second, we hypothesized that a comprehensive conceptual model would satisfactorily map the network of associations between emotion knowledge, theory of mind, and language ability. We predicted that both emotion knowledge and language would explain variance in, and wield positive direct effects on, theory-of-mind skills. The proposed conceptual model is presented in Figure 1.

## Materials and Methods

### Participants

The sample comprised 139 (71 girls) typically developing children. Participants’ age ranged from 24 to 47 months (*M* = 35.5 months; *SD* = 6.73). All children were native Italian speakers and were recruited through infant-toddler centers and kindergartens in North-Western Italy. They predominantly came from middle-class socioeconomic backgrounds. The study was conducted in conformity with the recommendations of the University of Milano – Bicocca Ethics Committee. Parental written informed consent was obtained for all participants in keeping with the Declaration of Helsinki.

### Measures

We adopted four measures: The Affective Knowledge Test (Denham, 1986); the Diverse-Desire Task (Wellman and Liu, 2004); the True-Belief Task (Wellman, 1991); and the Peabody Picture Vocabulary Test – Revised (Dunn and Dunn, 1981). The instruments were administered in counterbalanced order. Children were tested individually in a quiet room at their infant-toddler center or preschool.

#### Affective Knowledge Test (AKT)

The Italian validated version of the AKT (Camodeca and Coppola, 2010) was used to measure young children’s emotion knowledge. The first part of the test is designed to assess emotion recognition. The child is asked to verbally label four basic emotions (i.e., happiness, anger, sadness, and fear) depicted on faces (expressive task) and then to point to faces representing the emotions expressed verbally by the experimenter (receptive task). The second part of the AKT tests the child’s emotion situation knowledge. It consists of identifying the face that best depicts a character’s emotion as described in a brief story, in stereotypical situations (where most people identify with the story character’s emotion: stereotypical script task) and non-stereotypical situations (where the character experiences an emotion different to that likely to be felt by child, as previously reported by a caregiver in answer to a questionnaire; non-stereotypical script task). Children’s scores for each task ranged from 0 to 2: 0 for a completely inappropriate response; 1 for an incorrect response with the correct emotional valence; 2 for a correct response. Two composite scores were then calculated: emotion recognition, ranging from 0 to 16 (the sum of scores obtained in the expressive and receptive tasks), and emotion situation knowledge, ranging from 0 to 18 (the sum of scores obtained in the stereotypical and non-stereotypical script tasks). Raykov’s (1997) composite reliability for the global AKT scores was 0.76.

#### Diverse-Desire Task and True-Belief Task

We administered the Italian version of these tasks to assess the children’s theory of mind abilities. We adopted these two measures of theory of mind because their level of complexity was appropriate for the age groups under study, thus reducing the risk of obtaining random answers. The first task is designed to assess the child’s understanding that people may have desires that differ from their own. The second task is used to assess the understanding that other people can hold true beliefs. For each task, children scored 0 points for an incorrect answer and 1 point for a correct answer. Then, scores were summed so that overall scores ranged between 0 and 2. Raykov’s (1997) global composite reliability for the two dimensions of theory of mind was 0.69.

#### Peabody Picture Vocabulary Test – Revised (PPVT-R)

We adopted the Italian standardized version of the PPVT-R (Stella et al., 2000) to assess the children’s receptive vocabulary. The test consists of 180 cards of increasing complexity, each featuring four numbered illustrations. The standard procedure for the administration and scoring was as follows: the examiner called out a word and the child was asked to point to the matching illustration; the child scored 0 points for a wrong answer and 1 point for a correct answer. Raw unstandardized scores were used.

### Overview of Analyses

Prior to conducting the main analyses, the data were checked for univariate normality (i.e., distribution, kurtosis, and skewness). Mahalanobis’ distance (*p* < 0.001) was computed for the entire data set to identify any multivariate outliers. More specifically, kurtosis and skewness were calculated for the Affective Knowledge Test and Peabody Picture Vocabulary Test scores. In contrast, in relation to the theory of mind tasks (i.e., interval-type measures with few score categories), we only assessed the data to exclude the possibility that the distribution was heavily skewed. Data was found to be normally distributed, with skewness and kurtosis values falling within the accepted range of ±2 (George and Mallery, 2010). There were no missing values. Next, the main descriptive statistics and zero-order correlations were computed.

With regard to the conceptual model, emotion knowledge was treated as a latent endogenous variable comprising two domains (i.e., emotion recognition and emotion situation knowledge), while language and theory of mind were modeled as cumulative observed indicators. Age and gender were assessed as exogenous controlling factors modeled as wielding direct effects on all endogenous latent variables. In keeping with the literature, language and emotion knowledge were treated as covariates.

As is standard when conducting SEM (Kline, 2011; Pepe et al., 2017; Veronese and Pepe, 2018), the model’s goodness of fit with the data was assessed by calculating a set of indexes. These measured the overlap between the observed (S) and reproduced (Σ) matrices of covariance. Specifically, we calculated the following absolute and relative indexes: χ2 and normed-χ2 (NC), where a non-statistically significant χ2 value and NC values of under 2.0 indicate good fit (Hair et al., 2010); the root mean square error of approximation (RMSEA); normed fit index (NFI); non-normed fit index (NNFI); and comparative fit index (CFI). Thresholds for good model fit were: RMSEA <0.07 (Schermelleh-Engel et al., 2003), NFI >0.95, NNFI >0.95 (Marsh and Hau, 2014), and CFI >0.95 (Hu and Bentler, 1999). The maximum likelihood algorithm (Rhemtulla et al., 2012) was used to estimate the parameters for the structural model. In addition, we estimated confidence limits (MacKinnon et al., 2004) by applying Monte Carlo simulation and bootstrapping methods to a set of random samples (*k* = 500). We calculated given indirect effects for each of the k samples and the mean value for the selected pool of samples. All analyses were conducted using SPSS AMOS 23 software (Arbuckle, 2014).

## Results

Descriptive statistics and zero-order correlations for the variables under study are reported in Table 1. The correlational analysis revealed statistically significant positive associations among all variables. In particular, medium-sized correlations were found between language and both emotion knowledge/total score (*r* = 0.66, *p* < 0.001) and theory of mind scores (*r* = 0.32, *p* < 0.01). Similarly, the correlation between emotion recognition and theory of mind (*r* = 0.24, *p* < 0.01) was found to be positive and statistically significant. Finally, age was positively associated with all the measured abilities, with correlations ranging from 0.37 (theory of mind scores) to 0.65 (language ability).

**TABLE 1:** Means, standard deviations, and zero-order correlations among the study variables.

	***M***	***SD***	**1**	**2**	**3**	**4**	**5**	**6**	**7**
1. ER	9.49	4.03	–						
2. ESK	12.65	3.86	0.41^∗∗∗^	–					
3. EK (total)	22.14	6.64	0.85^∗∗∗^	0.83^∗∗∗^	–				
4. ToM	1.35	0.66	0.24^∗∗^	0.16^†^	0.24^∗∗^	–			
5. Language	24.14	14.38	0.55^∗∗∗^	0.56^∗∗∗^	0.66^∗∗∗^	0.32^∗∗∗^	–		
6. Age in months	35.47	6.73	0.51^∗∗∗^	0.55^∗∗∗^	0.63^∗∗∗^	0.37^∗∗∗^	0.65^∗∗∗^	–	
7. Gender	–	–	−0.18^∗^	0.037	–0.086	–0.033	–0.13	−0.077	–

*ER, emotion recognition; ESK, emotion situation knowledge; EK, emotion knowledge (total score); ToM, theory of mind. ^†^*p* = 0.050–0.070, ^∗^*p* < 0.05, ^∗∗^*p* < 0.01, and ^∗∗∗^*p* < 0.001.*

**FIGURE 2:**
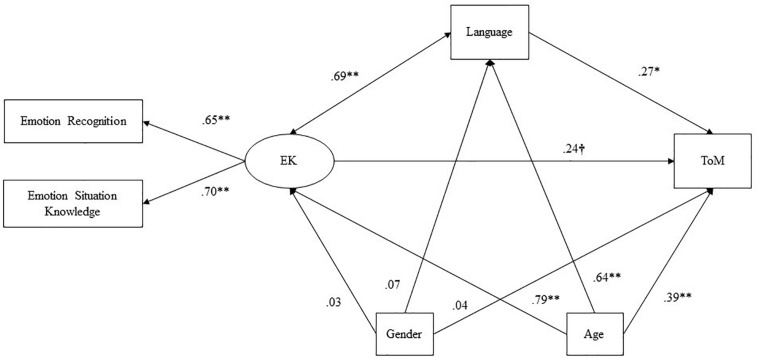
Results of the structural equation analysis. Standardized direct effects were reported. ^†^*p* = 0.069, ^∗^*p* < 0.05, and ^∗∗^*p* < 0.01.

The structural equation analysis showed that the empirical data fitted well with the conceptual model [χ2 = 7.91, *p* = 0.095; NC = 1.97, RMSEA = 0.079 90th CI (0.001–0.171), NFI = 0.965, NNFI = 0.982, CFI = 0.983], suggesting adoption of the model (see Figure 2) and analysis of its direct and indirect effects.

Analysis of direct effects revealed that emotion knowledge wielded a small-sized standardized positive effect on theory-of-mind scores, β = 0.24, *p* = 0.069, 95th CI [0.31–0.166]; the statistical significance of the effect was marginal. A similar (i.e., in terms of direction and magnitude) effect was found between language scores and theory of mind scores [β = 0.26, *p* = 0.040, 95th CI (0.002–0.025)]. In addition, the co-variation between children’s language scores and emotion knowledge revealed a strong association (ϕ = 0.69, *p* = 0.001) between the two constructs. With regard to participants’ demographic characteristics, the outcomes of the model suggested that gender had no significant direct effects on the other variables. In contrast, age played a more important role, wielding direct standardized positive effects on emotion knowledge [β = 0.81, *p* = 0.003, 95th CI (0.277–0.356)], language ability [β = 0.65, *p* = 0.004, 95th CI (1.23–1.51)] and theory of mind scores [β = 0.43, *p* = 0.006, 95th CI (0.028−0.043)].

## Discussion

The main purpose of the current study was to investigate the associations between emotion knowledge, theory of mind, and receptive language in 2- and 3-years-old children by testing a single comprehensive model. We obtained two main findings. First, we found statistically significant associations between emotion knowledge, theory of mind, and receptive language, even after controlling for age and gender. Second, a comprehensive structural model including emotion knowledge, language, and theory of mind proved to be an appropriate theoretical and conceptual structure, with stable and robust patterns of associations. These main findings will be now discussed in detail.

### The Associations Between Emotion Knowledge, Theory of Mind, and Language

We found statistically significant correlations among the investigated variables, even after controlling for the effect of age and gender. This result is in line with a great number of studies that have pointed up strong interdependence between these skills, including in young children (Denham, 1986; Cutting and Dunn, 1999; de Rosnay et al., 2004; Harwood and Farrar, 2006; Bulgarelli and Molina, 2016).

Positive associations emerged between language and both emotion knowledge and theory of mind skills. This finding is consistent with previous studies demonstrating that children’s language abilities represent a valuable asset for the development of both cognitive and emotional skills (Milligan et al., 2007; Imuta et al., 2016; Conte et al., 2018). This is partly because acquiring language enables children to put desires, thoughts, and emotions into words. The process of language acquisition begins early, as demonstrated by the onset and use of psychological lexicon at around 24 months (Harris et al., 2016). Attaining this developmental milestone has a hugely positive effect on the child’s wellbeing, as becomes extremely clear when the child experiences linguistic delays or impairments. Indeed, early language difficulties cause communication problems, which in turn can lead the child to avoid social exchanges right from the early years (Rieffe and Wiefferink, 2017).

### The Direction of Effects in the Structural Model

A further key finding was that emotion knowledge, theory of mind, and language were satisfactorily represented by a single comprehensive model. Such an outcome suggests that these skills are components of an integrated system: As mentioned above, language and social cognition have powerful interconnections and progress in one area is crucial for the ongoing development of the other. The same model also yielded significant outcomes when assessed with older – preschool and school age – children (Bulgarelli and Molina, 2016; Grazzani et al., 2018). To our knowledge, no research to date has examined these same variables in young children within a single comprehensive framework. Hence, our study reveals that from the early years, receptive language, and the capacity to recognize emotions and their contextual cues work in synergy with the ability to read others’ minds.

Furthermore, emotion knowledge and language both made a relevant contribution to explaining variance in theory of mind performance. While language wielded a statistically significant effect on theory of mind, emotion knowledge had only a marginal effect on it. However, the magnitude of the effect of emotion knowledge on theory of mind performance suggested that a larger sample size would likely have yielded a statistically significant effect (see Moore et al., 2012; Sullivan and Feinn, 2012). This association between children’s emotion knowledge and the ability to reason about others’ minds bears out the outcomes of longitudinal studies with children slightly older than those who participated in our study (Hughes and Dunn, 1998; Eggum et al., 2011; O’Brien et al., 2011). This result may be due to the different developmental trajectories of emotion knowledge and theory of mind. Although they are both social cognition skills, children recognize and understand emotions before they learn to explicitly attribute mental states to other people (O’Brien et al., 2011). This developmental difference may be particularly evident in first years of life, when these abilities are being acquired. Recognizing basic emotions in oneself and others may help young children to understand that people have inner states, and gradually to formulate a theory about others’ minds. In fact, as argued by Eggum et al. (2011), it is possible that older children may benefit from gains in theory of mind skills enabling them in turn to better understand other people’s emotions. The outcomes of this study represent fresh evidence for this direction of influence between emotion knowledge and theory of mind performance in younger children. Future research might be focused on investigating whether the effect is reversed or becomes reciprocal as children progress from toddlerhood to preschool age. With regard to language abilities, these were found to have statistically significant and positive associations with both emotion knowledge and theory of mind skills. Specifically, children’s emotion recognition and situational knowledge co-varied with language, lending support to the current literature that emphasizes the reciprocal effects between these abilities in young children. Indeed, our findings are similar to those obtained by Ornaghi et al. (2019), whose SEM suggested that emotion knowledge co-varied with language skills. Given that the children in the sample all fell within the same limited age range, it appeared that improvement in one area should lead to a corresponding gain in the other.

Concerning the association with theory of mind skills, our findings confirmed previous research attesting the direct effects of children’s language abilities on their capacity to reason about others’ mental states (Astington and Jenkins, 1999; Ruffman et al., 2002; Milligan et al., 2007; San Juan and Astington, 2017). We know from the literature that language abilities are a crucial tool for children in their everyday lives and benefit their overall development in multiple ways. For instance, research on young children has shown that more advanced language skills predict greater concern and less disregard for others (Rhee et al., 2013). Furthermore, children younger than 4-years-old with greater receptive language skills tend to behave more prosocially toward their peers (Conte et al., 2018). It is likely that these behavioral outcomes may be due to the primary effect of language on theory of mind skills. In fact, as mentioned above, language abilities help to practice one’s communication skills and encourage reasoning about people’s thoughts and behaviors.

The key role played by language in children’s development was further confirmed by the indirect effects that emerged from the structural equation analyses. Our findings suggest that emotion knowledge had indirect effects on theory of mind via language ability. This is in line with in the outcomes of previous studies. In fact, Bulgarelli and Molina (2016) reported that language had both direct and indirect effects on preschool children’s theory of mind ability. Similarly, Grazzani et al. (2018) found that children’s linguistic skills mediated the relationship between emotion understanding and theory of mind performance.

Last but not least, the results of the present study are in line with literature suggesting that emotion knowledge, language, and theory of mind skills follow an age-related developmental trajectory throughout early childhood. In fact, age was found to have direct effects on all the variables under study. Multiple studies have shown that older children are better than younger children at labeling facial expressions and identifying emotions across different situations. For instance, Sette et al. (2015) found that 2- and 3-year-old children obtained lower scores for both emotion recognition and stereotypical situation knowledge than did 4- and 5-year-old children. Theory of mind performance and language ability also vary as a function of age. Wellman (2014, 2018) has theorized that children’s understanding of people’s minds progresses through a fixed series of developmental milestones, from understanding desires and to appreciating concealed emotions. Such social cognitive gains may be related to concurrent gains in language ability, which also improves with age, as attested by the appearance of a psychological lexicon as soon as children begin to talk.

### Educational Implications and Limitations

In sum, the current findings suggest that language and emotion knowledge are crucial factors in young children’s developing of theory of mind, and this is already the case as early as toddlerhood. To our knowledge, this is the first study to date to have investigated the associations between these variables in a single model focused on this young age range. Despite the strengths of this contribution, some shortcomings need to be acknowledged.

First, the model was tested within a cross-sectional research design rather than with a longitudinal design with the power to clarify whether the variables continue to play the same key role later in development. Second, only two measures of theory of mind were administered in place of a complete battery of tests that might have yielded more robust information about the children’s ability to understand the minds of others. However, our results are quite encouraging and suggest that emotion knowledge and language affect early-appearing theory-of-mind skills such as the understanding of desires and true beliefs.

Altogether, these findings bear key educational implications. In fact, they suggest the importance of fostering children’s language acquisition from the earliest years of life with a view to boosting their early development of social cognition skills. In this regard, recent studies have pointed up the value of conversational exchanges about internal states (the children’s own and those of others) – even in the infant-toddler center setting – for the development of social and emotional abilities (Agliati et al., 2015; Giménez-Dasí et al., 2015; Grazzani et al., 2016b; Ornaghi et al., 2017). The presence of an adult and other peers during joint activities, such as listening to books together and talking about story characters’ mental states (e.g., emotions and desires), offers children the opportunity to reason about and discuss others’ inner states. Such conversations may foster children’s ability to recognize emotions and understand their causes, as well as to grasp their connection with desires and beliefs. For instance, Grazzani et al. (2016b) found that stimulating conversations about mental states in young children attending nursery school had positive effects on their emotion knowledge and theory of mind skills which remained stable over time. In turn, improved social cognition is associated with multiple positive outcomes, such as good peer relationships, popularity with peers, prosocial behaviors (Imuta et al., 2016; Ornaghi et al., 2016), school readiness, and adjustment (Wang, 2015; Brock et al., 2019).

Given that young children begin to develop all these skills in the earliest years of life, parents and educators need to be aware of the importance of promoting children’s emotion knowledge and language competence from toddlerhood. However, many adults are not sufficiently informed about, or trained in, using conversational approaches to promoting young children’s social cognition skills (Grazzani et al., 2011). When adults are appropriately trained, they can play a key role in enhancing children’s mindreading abilities and consequently their positive social behaviors.

## Data Availability

The raw data supporting the conclusions of this manuscript will be made available by the authors, without undue reservation, to any qualified researcher.

## Ethics Statement

The studies involving human participants were reviewed and approved by University of Milano – Bicocca Ethics Committee. Written informed consent to participate in this study was provided by the participants’ legal guardian/next of kin.

## Author Contributions

EC contributed to designing the study, collecting, interpreting, and discussing the data, and writing and revising the manuscript. VO contributed to designing the study, interpreting and discussing the data, and drafting and revising the manuscript. IG made a key contribution to designing the study, interpreting and discussing the data, and drafting and revising the manuscript. AP made a key contribution to analyzing, interpreting the data, and revising the manuscript. VC contributed to discussing the findings and revising the manuscript.

## Conflict of Interest Statement

The authors declare that the research was conducted in the absence of any commercial or financial relationships that could be construed as a potential conflict of interest.
